# Supplementary material for: A Humanized Yeast Phenomic Model of Deoxycytidine Kinase to Predict Genetic Buffering of Nucleoside Analog Cytotoxicity
Source: Genes (Basel). 2019 Sep 30;10(10):770. doi: 10.3390/genes10100770 (PMC6826991; doi:10.3390/genes10100770)
Supplement: Supplementary file 1 [file genes-10-00770-s001.zip › Additional_Files/Additional_File7_GOTermHeatmaps.zip!/Additional_File7_GOTermHeatmaps/cytosolic_ribosome.pdf]

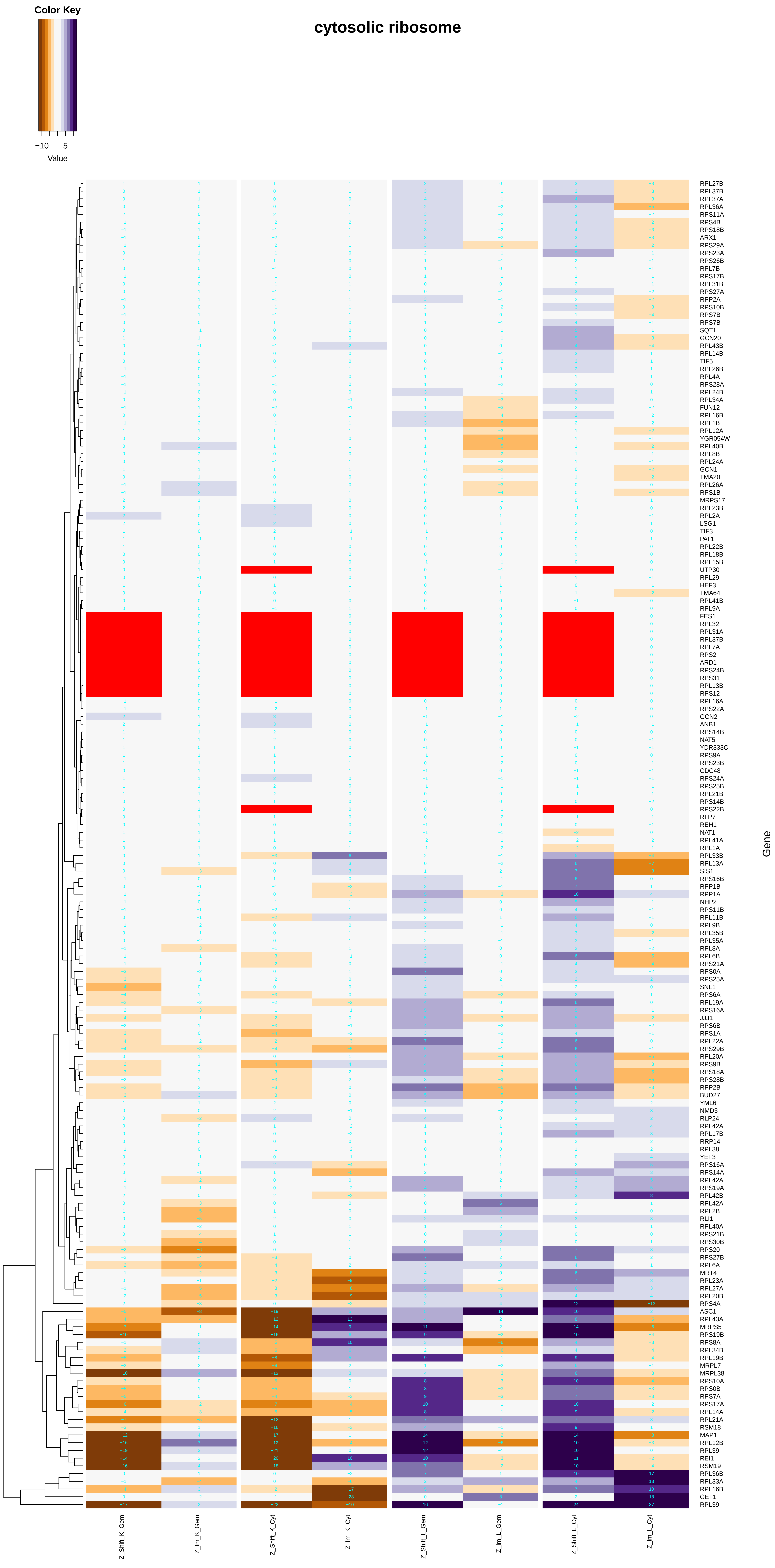
Color Key
−10
5
Value
cytosolic ribosome
RPL27B
RPL37B
RPL37A
RPL36A
RPS11A
RPS4B
RPS18B
ARX1
RPS29A
RPS23A
RPS26B
RPL7B
RPS17B
RPL31B
RPS27A
RPP2A
RPS10B
RPS7B
RPS7B
SQT1
GCN20
RPL43B
RPL14B
TIF5
RPL26B
RPL4A
RPS28A
RPL24B
RPL34A
FUN12
RPL16B
RPL1B
RPL12A
YGR054W
RPL40B
RPL8B
RPL24A
GCN1
TMA20
RPL26A
RPS1B
MRPS17
RPL23B
RPL2A
LSG1
TIF3
PAT1
RPL22B
RPL18B
RPL15B
UTP30
RPL29
HEF3
TMA64
RPL41B
RPL9A
FES1
RPL32
RPL31A
RPL37B
RPL7A
RPS2
ARD1
RPS24B
RPS31
RPL13B
RPS12
RPL16A
RPS22A
GCN2
ANB1
RPS14B
NAT5
YDR333C
RPS9A
RPS23B
CDC48
RPS24A
RPS25B
RPL21B
RPS14B
RPS22B
RLP7
REH1
NAT1
RPL41A
RPL1A
RPL33B
RPL13A
SIS1
RPS16B
RPP1B
RPP1A
NHP2
RPS11B
RPL11B
RPL9B
RPL35B
RPL35A
RPL8A
RPL6B
RPS21A
RPS0A
RPS25A
SNL1
RPS6A
RPL19A
RPS16A
JJJ1
RPS6B
RPS1A
RPL22A
RPS29B
RPL20A
RPS9B
RPS18A
RPS28B
RPP2B
BUD27
YML6
NMD3
RLP24
RPL42A
RPL17B
RRP14
RPL38
YEF3
RPS16A
RPS14A
RPS19A
RPL42B
RPL42A
RPL2B
RLI1
RPL40A
RPS21B
RPS30B
RPS20
RPS27B
RPL6A
MRT4
RPL23A
RPL27A
RPL20B
RPS4A
ASC1
RPL43A
MRPS5
RPS19B
RPS8A
RPL34B
RPL19B
MRPL7
MRPL38
RPS10A
RPS0B
RPS7A
RPS17A
RPL14A
RPL21A
RSM18
MAP1
RPL12B
RPL39
REI1
RSM19
RPL36B
RPS33A
RPL16B
GET1
RPL39
Gene
Z_Shift_K_Gem
Z_Im_K_Gem
Z_Shift_K_Cyt
Z_Im_K_Cyt
Z_Shift_L_Gem
Z_Im_L_Gem
Z_Shift_L_Cyt
Z_Im_L_Cyt
